# Neighborhood Environment and Self-Rated Health among Adults in Southern Sri Lanka

**DOI:** 10.3390/ijerph6082102

**Published:** 2009-07-29

**Authors:** Bilesha Perera, Truls Østbye, Chandramali Jayawardana

**Affiliations:** 1 Faculty of Medicine, University of Ruhuna, Galle, Sri Lanka; 2 Duke Medical Center, Duke University, Durham, NC 27710, USA; E-Mail: ostby001@mc.duke.edu; 3 Faculty of Applied Sciences, Sabaragamuwa University of Sri Lanka, Belihuloya, Sri Lanka; E-Mail: jayawardanack@yahoo.com

**Keywords:** environment, neighborhood, stressors, self-rated health, Sri Lanka, ecology

## Abstract

The prevalence of different neighborhood environmental stressors and associations between the stressors and self-rated health are described in a representative sample of 2,077 individuals, aged 18–85 years, in southern Sri Lanka. Mosquito menace (69.4%), stray dog problems (26.8%), nuisance from neighbors (20.3%), and nuisance from drug users (18.7%) were found to be the most prevalent environmental stressors. None of the stressors investigated were associated with self-rated *physical* health, but nuisance from neighbors, nuisance from drug users, shortage of water and having poor water/sewage drainage system were associated with self-rated *mental* health among the respondents.

## 1. Introduction

The living environment plays a vital role in determining health [1–3]. Individuals living in poor neighborhood environments tend to have higher morbidity and mortality compared to those living in environmentally sound neighborhoods [3–5]. Various measures have been taken to improve the environment, for its own sake, and to improve public health. Such measures include the establishment of environmental protection acts, public education, and support for research on environment and health [1,5–7]. The Sri Lankan government prepared a national environment action plan in 1991 and implemented a set of policy measures for environmental health shortly thereafter [8]. These actions show commitment to the protection and improvement of its environment for a healthy nation. In addition, the Ministry of Healthcare and Nutrition in Sri Lanka has long been providing technical guidance to various organizations and to other ministries (e.g., Urban Development and Education), to strengthen the environmental health conditions in the country [9]. Further, a large number of non-governmental organizations active in Sri Lanka also work to make the environment healthier, for example through waste disposal, sanitation and water supply [10–12].

In Sri Lanka, studies of the spread of mosquito-born diseases (i.e., malaria, dengue and filariasis) and poor neighborhood conditions have found an association between these. Important environmental factors that permit the spread of such diseases include deforestation, dumping of waste in open places, inadequate cleaning facilities, and lack of sustainable mechanisms available to eliminate mosquito breeding sites [10,13,14]. These studies have highlighted the need for cost effective environmental prevention measures of these diseases, not only because of the adverse health effects, but also because the economic burden the country as a whole suffers due to the high cost involved in treating these disease conditions. In Sri Lanka, despite control measures, mosquito-borne diseases remain a major public health issue [10,11,13].

Studies of sanitation and availability of safe drinking water conducted in the country have provided insights into the adverse effects of poor sanitation and unsafe drinking water on health, and the impact of human behavior on such environmental issues. It has been shown that the nature of water supply and sanitation conditions in some parts of the country are associated with infant, child and maternal health problems such as diarrhea and dental fluorosis [15–17]. Moreover, the public’s passive attitude towards sanitary improvements and funding problems have hampered the progress of many community-based water supply and sanitation projects implemented by both the government and non-governmental organizations [18]. The National Water Supply and Drainage Board in Sri Lanka is the principal agency responsible for water supply and sanitation. According to the Board, there are regional differences in providing water and sanitation facilities to the nation. They have adopted “people-centered” and “demand driven” approaches in their rural water supply and sanitation projects [12]. The 2004 tsunami badly affected the Board’s activities in the Southern Province, which are still in a recovery process. The key challenge the Board faces is lack of resources. Further, many government and non-governmental departments and organizations including the departments of Health and Education, Urban Development Authority, and provincial and local councils have the responsibility of creating a healthy living environment for the people. But responsibilities and tasks were not clearly identified nor agreed upon by these institutions [11, 12]. As a result, each agency’s responsibilities are unclear, resulting in poor outcomes. Proper coordination between these units, community participation and political commitment are needed to implement sustainable and effective measures to protect the neighborhood environment.

Population growth, lack of resources and urbanization have also contributed to increased environmental problems in the country [19]. Further, air pollution, mismanagement of waste, and lack of recreation facilities have led to an increased susceptibility to environmentally-induced respiratory and cardiovascular diseases. Studies conducted on the effects of air pollution on health have shown that people living in highly populated industrial areas compared to people living in other areas have increased rates of respiratory problems [20,21]. Most government-run municipal solid waste management programs deal only with the collection and dumping of waste, not its treatment or disposal [11], thus also creating environmental and health hazards.

Despite their public health relevance and importance, little research beyond mosquito-borne diseases has been conducted on neighborhood environmental stressors and health. Issues such as air and sound pollution, recreational facilities, neighborhood quality, sanitation, and garbage disposal have received little attention. A better understanding of prevailing neighborhood environmental stressors, the relationship between these stressors and health, and the effectiveness of healthy environmental interventions are essential to formulating policies, plans and actions to improve the quality of neighborhood environments which subsequently enhance the nation’s health.

The objectives of this study were to investigate: 1) the prevalence of reported neighborhood environmental stressors, and 2) associations between self-rated health and such stressors in a representative sample of southern Sri Lankans. Opinions about the services provided by public servants and institutions responsible for environmental sanitation and health were also elicited and examined.

## 2. Participants and Method

### 2.1. Participants

The study was conducted in Southern Sri Lanka in 2006–2007. A cross-sectional survey design was used. The target population was all adults age 18 years or above, living in the three districts: Galle, Matara and Hambantota, in the Southern Province. Approximately 2,000 subjects were surveyed. Non-response rate was less than 5%. Stratified proportional quota sampling was used. The sample was selected to achieve equal proportions of men and women from all age categories. The sample size for each district was proportional to its population size. Approximately 43% of population in the Southern Province live in Galle, 33% in Matara and 24% in Hambantota [22]. The Galle district is divided into 18 Divisional Secretariat (DS) divisions, Matara into 16 and Hambantota into 11 DS divisions. Three DS divisions from Galle, two from Matara and two from Hambantota were selected to represent major characteristics of people in southern Sri Lanka. Each of these DS divisions has several “Gramaseva Niladhari Divisions” (GND). Two or three GNDs were selected from each DS division for this survey. GNDs were selected so as to represent both urban and rural communities. A household in a chosen GND was selected randomly and then, subsequent households were identified by following a random direction from the previous household until the required number of households was surveyed. We surveyed all members aged 18 or older who were in the home at the time of the survey. This procedure was continued until the desired sample size was achieved. The final sample consisted of 2,091 participants, representing Galle [918 (43.9%)], Matara [647 (30.9%)] and Hambantota [526 (25.2%)].

### 2.2. Methods

Data were collected from the participants using interviewer-administered, anonymous questionnaires. In the questionnaire, questions on neighborhood environment included a wide range of environmental characteristics related to health among adults in the Southern Province (Table 1). To identify the most common local neighborhood environmental problems and stressors for inclusion in the questionnaire, we solicited opinions from three experts (an Environmental Scientist, Community Physician and a Medical Officer of Health (MOH)). In addition, a literature review was conducted.

Self-rated physical health and mental health were assessed using the questions “*How would you rate your physical health in general?”* and “*How would you rate your mental health in general?”* Five possible responses to each question were dichotomized by assigning “0” to those who answered very good or good and “1” to those who answered moderate, poor or very poor. The questionnaire was first developed in English, translated to Sinhalese (the native language) and finally back translated to English–only the Sinhalese version was administered to participants. The survey was pilot tested (n = 25) and refined. The final questionnaire took approximately 40 minutes to complete and was distributed by a group of 12 trained research assistants (science or arts degree graduates). This study was approved by the Ethics Review Committee, Faculty of Medicine, Galle, and by the Institutional Review Board of Duke University Medical Center, Durham, North Carolina, USA.

### 2.3. Data Analysis and Statistical Methods Used

The data were entered into Microsoft Excel and analyzed using the Statistical Package for the Social Sciences (SPSS) version 14. Simple descriptive statistics were used to examine the variables. The associations between each neighborhood stressor and moderate to very poor self-rated physical and mental health were estimated using logistic regression. The multivariate models were adjusted for age, sex and income level because evidence suggests that people in different sex, age and income categories interpret or perceive self-rated health differently [23,24].

## 3. Results

After consistency checking, the analysis sample included 2,077 subjects, of which 44.8% (*n =* 931) were males. The mean age was 40.16 years (*SD =* 15.7, range 18 to 85 years). Nearly 12% of the participants did not report family income. The estimated average family income in the participant’s area was used as an approximation for those who did not report family income. Of the total, 29% were from lower (monthly family income <US$ 50), 65.2 % from middle (US$ 51–300) and 5.8% from upper (>US$ 300) income brackets.

### 3.1. Neighborhood Environmental Stressors

Mosquito menace, stray dogs, nuisance from neighbors and nuisance from drug users were common environmental stressors reported by the respondents (Table 1). Among them, mosquito menace was the most frequently reported environmental stressor.

Overcrowding, ventilation problems, shortage of water and garbage disposal problems were also identified as neighborhood environmental stressors by a significant proportion of respondents. When analyzing data separately for each of the stressors investigated, it was observed that the proportion of participants who reported having been exposed to the stressor was slightly higher among those with negative mental health status compared to those with negative physical health status, although no significant differences were found.

### 3.2. Self-Rated Health and Neighborhood Environmental Stressors

The adjusted associations between each neighborhood stressor and moderate to very poor self-rated health are shown in Table 2. There were no significant relationships between the neighborhood environmental stressors investigated and moderate to poor self-rated *physical* health. However, there was an association with self-rated *mental* health. Individuals living in neighborhoods with nuisance from neighbors, nuisance from drug users, shortage of water and poor sewage/drainage system were more likely to report moderate to very poor self-rated mental health. Although the mosquito and stray dog problems were the most prominent environmental stressors reported, the two stressors were not associated with self-rated physical or mental health.

### 3.3. Opinions on Environmental Health Service Providers

The participants were asked to give their opinions on the services provided by the government servants/institutes with responsibility for improving environmental health conditions in their respective areas. Bivariate analysis of the opinions expressed by the participants and exposure to important environmental stressors that these servants/institutes are directly working on are presented in table 3.

The Public Health Midwife (PHM) has the responsibility of advising and improving sanitary conditions of mothers, infants and other young children and the Public Health Inspector (PHI) is responsible for improving neighborhood environmental conditions such as safe drinking water, waste disposal, elimination of mosquito breeding sites, and canine immunization [9]. The local administrative bodies (municipal councils and Predeshiya Sabhas) are responsible for waste disposal and bio-safety in their respective areas [11].

About half of the participants felt that the services provided by the PHM were satisfactory. About one fourths were satisfied with the services provided by the PHIs. About 39% of the participants expressed satisfaction with the services provided by the local administrative authorities in areas where they live.

Overall, nearly one fifths of the participants reported having “no idea about” the services provided by the PHM and local government authorities, and one fourths of the participants reported the same for the services provided by the PHI. A higher percentage of participants who reported having had exposed to mosquito and poor sewage/water drainage problems compared to non-exposed, expressed dissatisfaction with the services provided by the PHM (*p* < 0.05). A higher percentage of participants who reported having had exposed to mosquito, garbage disposal and poor sewage/water drainage problems compared to non-exposed, expressed dissatisfaction with the services provided by the PHI (*p* < 0.05).

Those who had exposed to garbage disposal and mosquito problems were more likely than non-exposed to express dissatisfactory views about the services provided by the local government authorities (*p* < 0.05).

## 4. Discussion

This study examined the prevalence of neighborhood environmental stressors, and associations between these stressors and self-rated physical and mental health among people in Southern Sri Lanka. In general, mosquito and stray dog problems, and nuisance from neighbors and drug users were the most commonly reported environmental stressors. Overcrowding, ventilation and garbage disposal problems were also identified as neighborhood stressors. Given that about 70% of the respondents in our study reported mosquitoes as a neighborhood stressor, periodic evaluations of existing mosquito control measures are needed to inform, and if necessary, adjust, strategies for community planning and development. Our findings also highlight the need to encourage research on such issues and effectively use such data to design and plan environmentally sound neighborhoods in the country.

Self-rated health is a strong and independent predictor of mortality and morbidity [25], and considered as a valid and robust measure of general health status. Although self-rated mental health and self-rated physical health have not been investigated as extensively as self-rated health, such measures are increasingly being used in health related research [26,27]. In this study, self-rated physical and mental health were considered separately. Self-rated moderate to very poor mental health was associated with perceived nuisance from neighbors, nuisance from drug users, shortage of water, and poor sewage/drainage system in the area. Nuisance from drug users seems to be the most prominent neighborhood stressor associated with poor mental health. Alcohol is the most prevalent drug used by people in Sri Lanka, and illegally produced alcoholic beverages which have a higher alcohol concentration than legally produced alcoholic beverages are available in most parts of the country. Living near individuals who abuse such substances may be a proxy for a number of neighborhood problems. Some of those who use illegal alcoholic beverages tend to engage in violent behavior impacting their neighbors. Shortage of water is another important environmental stressor. A considerable proportion of those living in Southern Sri Lanka live in costal areas where drinking water has to be brought from far away or purchased at higher prices. Time and energy spent on getting water may therefore cause psychological stress. This investigation did not find associations between moderate to very poor self-rated physical health and our selected neighborhood stressors. So, perceived nuisance from neighbors, nuisance from drug users, shortage of water and poor sewage/drainage system are likely important risk factors of psychological health of adults in Southern Sri Lanka. Although some studies have been conducted on waste disposal and water quality, the authors are aware of no studies relating to other important environmental stressors such as a neighborhood’s social and physical stressors. More research in these areas is warranted.

There may be different mechanisms through which the environmental stressors affect perceived health. As suggested by Leslie and Cerin [28] perceived satisfaction with neighborhood conditions may mediate the associations between self-report neighborhood environmental stressors and measures of mental health in this adult population. A large number of studies have been conducted in developed countries on neighborhood environmental conditions and perceived health. Multilevel studies which consider both individual factors and contextual factors in relation to health have indicated significant associations between neighborhood economic characteristics, neighborhood social environment, neighborhood physical environment, neighborhood amenities and health in general [5,29,30]. It is unclear why only mental health was related to neighborhood environmental stressors in this study, but it is possible that people tend to report poor mental health more often than poor physical health because a significant proportion of people in the study area had been affected by the 2004 tsunami tragedy.

Most participants expressed “dissatisfaction with” or “no idea of” services provided by the areas’ public servants who have the responsibility of improving environmental conditions. Persons who reported exposure to environmental stressors were seems to be more likely than others to blame these service providers for poor conditions found in their living environments. This observation could be due in part to a misperception that these public servants have the sole responsibility of creating a healthy environment. This is one of the major barriers to implementing a sustainable waste management system in Sri Lanka [11]. These concerns need to be considered in the context of national planning. Given that psychological health problems in the country are on the rise [9], and that social and physical structures and functions in the country are rapidly changing, the results of this study highlight the need to reconsider national strategies for environment and health.

Limitations of this study include its cross sectional nature, not allowing us to assess the causal direction of any associations observed. It is less likely that poor self reported health leads to poor perceived environmental conditions than *vice versa* [4,5,31], but a third, underlying factor such as income or wealth may lead to both. Income data were missing for a sizeable number of participants. The analyses were also limited by the number of neighborhood stressors we investigated. Housing conditions, social capital, degree of urbanization, and size of the population are some of the environmental features that could have been included. We did not analyze data by urban versus rural residence, as the urban/rural demarcations made decades ago are no longer valid given the significant changes in habitation and social structure that have occurred since then. Both neighborhood stressors and health are based on self-report rather than objective assessments, and collected from the same group of participants. Although objective measures of some of the neighborhood factors would have been helpful, the *perception* of such stressors is also important. Longitudinal and more in-depth investigations are needed to identify reciprocal and reinforcing relationships between environmental stressors and health. Nevertheless, the relatively comprehensive battery of questions relating to environmental stressors, the large catchment area and the large sample size representative of all three districts in the Southern Province lend strength to the study.

## 5. Conclusions

Certain poor neighborhood environmental conditions, in particular mosquito and stray dog problems, nuisance from neighbors and drug users, and non-availability of water were prevalent among adults in Southern Sri Lanka, and most of these conditions were related to their psychological health. Most of the participants were either “not satisfied with” or “had no idea about” services provided by public servants who have responsibilities of improving environmental conditions in the area. Factors that limit these public servants from being effective public health workers need to be investigated. More ecological research is needed to support appropriate policy, planning and design for an environment that can better ensure the population’s physical and mental health.

## Figures and Tables

**Table 1.** Neighborhood environmental stressors by perceived health status.

**Environmental Stressor**	**Number (%) (N = 2077)**	**Number and percentage of participants reporting exposure to the stressor**	
		**Moderate to very poor mental health(n= 533)**	**Moderate to very poor physical health(n= 738)**

Nuisance from neighbors	422 (20.3%)	153 (20.7%)	127 (23.8%)
Nuisance from drug users	388 (18.7%)	144 (19.5%)	130 (24.4%)
Overcrowding/poor ventilation	214 (10.3%)	80 (10.8%)	61 (11.4%)
Shortage of water	300(14.4%)	108 (14.6%)	93 (17.4%)
Garbage disposal problems	278 (13.4%)	92 (12.5%)	65 (12.6%)
Mosquito problem	1,442 (69.4%)	531 (72.0%)	388 (72.8%)
Stray dog problem	557 (26.8%)	227 (30.8%)	167 (31.3%)
Nuisance from noise	74 (3.6%)	26 (3.5%)	19 (3.6%)
Poor water/sewage drainage system	132 (6.4%)	48 (6.5%)	37 (6.9%)

**Table 2.** Relationship between neighborhood environmental stressors and moderate to very poor physical and mental health: multivariate models adjusted for age, sex and income level (Odds ratios (and 95% CI); significantly increased odds of moderate to very poor health indicated with *).

**Neighborhood stressor**	**Moderate to very poor Physical Health OR (95% CI)**	**Moderate to very poor Mental Health OR (95% CI)**

Nuisance from neighbors	1.21 (0.92 to 1.58)	1.54 (1.16 to 2.03)*
Nuisance from drug users	1.23 (0.93 to 1.61)	1.75 (1.32 to 2.31)*
Overcrowding/poor ventilation	1.17 (0.81 to 1.68)	1.29 (0.87 to 1.89)
Shortage of water	1.17 (0.85 to 1.60)	1.66 (1.21 to 2.29)*
Garbage disposal problems	0.88 (0.64 to 1.19)	0.97 (0.70 to 1.34)
Mosquito problem	0.99 (0.76 to 1.28)	0.97 (0.73 to 1.28)
Stray dog problem	1.16 (0.91 to 1.48)	1.19 (0.92 to 1.54)
Nuisance from noise Poor	0.81 (0.44 to 1.46)	0.93 (0.49 to 1.76)
water/sewage drainage system	1.09 (0.70 to 1.69)	1.64 (1.05 to 2.56)*

**Table 3.** Respondents’ opinions about environmental service providers in the area by exposure to some selected environmental stressors (N = 2077).

**Environmental Stressor [exposed (yes) or not exposed(no)]**		**Are you satisfied with the services provided by**								
		**Midwife?**			**PHI?**			**Local government body?**		

		**yes**	**no**	**no idea**	**yes**	**no**	**no idea**	**yes**	**no**	**no idea**

**Garbage**	yes	51.1%	31.7%	17.2%	20.2%	56.8%	23.0%	29.5%	51.1%	19.4%
	No	50.4%	28.6%	21.0%	23.4%	49.0%	27.6%	40.5%	40.5%	19.0%
**Mosquito**	yes	49.3%	31.6%	19.1%	21.1%	54.6%	24.3%	38.5%	44.7%	16.8%
	No	53.6%	23.0%	23.4%	27.3%	39.6%	33.1%	40.0%	35.7%	24.3%
**Stray dog**	yes	51.9%	31.2%	16.9%	23.3%	50.4%	26.3%	42.4%	43.6%	14.0%
	No	50.0%	28.2%	21.8%	22.9%	49.9%	27.2%	37.8%	41.3%	20.9%
**Poor drainage**	yes	37.1%	42.4%	20.5%	17.4%	55.3%	27.3%	37.1%	43.2%	19.7%
	No	51.5%	28.1%	20.4%	23.3%	49.8%	26.9%	39.1%	41.9%	19.0%
